# The role of objectification in young men’s perpetration of intimate partner violence

**DOI:** 10.1371/journal.pone.0313016

**Published:** 2024-11-08

**Authors:** Adriana Vargas Sáenz, Nick Haslam

**Affiliations:** Melbourne School of Psychological Sciences, The University of Melbourne, Parkville, Australia; University of Cagliari: Universita degli Studi Di Cagliari, ITALY

## Abstract

Theorists have argued that objectification is implicated in men’s violence against women. Growing correlational and experimental evidence supports this claim. However, little research has studied the link between objectification and violence perpetrated by intimate partners. Three studies examined this link in relation to several forms of violent behavior. Study 1 (*N* = 215) found that men who implicitly associated women with objects were more likely to perpetrate sexual and physical violence against their female romantic partner, independent of their levels of hostile sexism. Study 2 (*N* = 325) replicated this finding but examined automatic associations with men’s intimate partners rather than women as a class. Greater implicit objectification was again associated with self-reported physical violence and with a behavioral proxy measure of aggression among participants who responded most strongly to an experimental provocation. Study 3 (*N* = 192) manipulated objectification by inducing a physical appearance-focus mindset and found that the manipulation increased men’s tendency to respond violently toward their partner. By implication, objectification appears to play a significant role in facilitating men’s violence in romantic relationships.

## Introduction

Male violence is a pervasive part of many women’s lives. Violence against women, defined by the United Nations as “any act of gender-based violence that results in, or is likely to result in, physical, sexual, or mental harm or suffering to women, including threats of such acts, coercion or arbitrary deprivation of liberty, whether occurring in public or in private life” [1], is recognized as an urgent global public health and human rights issue. The harm it inflicts is compounded by the frequency with which it is perpetrated by women’s intimate partners.

Intimate partner violence (IPV) is “any behavior within an intimate relationship that causes physical, psychological or sexual harm to those in the relationship” [2]. It includes physical and sexual violence and psychological abuse, as well as harassment, stalking, reproductive coercion, financial and social abuse, and image-based abuse. It often occurs in private settings and is likely to be under-reported, making estimates of its true prevalence and impact uncertain and probably conservative. Nevertheless, recent global estimates indicate that 30% of women have experienced physical and/or sexual violence at the hands of their male intimate partners in their lifetime [3]. As many as 58% of all murders of women and girls are committed by someone close to them [4].

Understanding the factors contributing to IPV perpetration by heterosexual men is a pressing task. Researchers have identified a wide range of risk factors at individual, interpersonal, institutional, and cultural levels of analysis and estimated the magnitude of their impacts [5, 6]. A vast literature has identified demographic, personality, attitudinal, developmental, and substance use history factors at the individual level, relationship quality factors at the interpersonal level, and cultural, socioeconomic, adversity, and inequality-related factors at the societal level.

One factor identified by feminist researchers and scholars is the tendency for women to be viewed or treated as objects, whether by locating their value in their physical appearance, perceiving them as possessions, or denying them agency and autonomy [7–12]. These critics argue that treating women as objects facilitates violence by representing them as less than fully human. Theoretical arguments such as these have been influential and popular, but until recently they have rarely been examined empirically.

New lines of research have begun to examine the causes and consequences of objectification as a psychological process [13]. Researchers typically conceptualize objectification in one of two inter-related ways, either as a focus on a person’s physical appearance at the expense of their other qualities or a failure to perceive attributes that differentiate humans from non-humans. On the latter conceptualization, objectification is akin to dehumanization: a denial of human characteristics that distinguish us from objects, such as emotions, personality, or individuality.

Studies adopting the former view of objectification have examined the effects of appearance focus on person perception [14, 15]. Those adopting the dehumanization view examine the extent to which some social targets are seen as lacking specific features of personhood such as mental states [16–20] or particular human traits, often divided into those that are essentially human in contrast to inanimate objects (Human Nature) and those that are unique to humans in contrast to other animals (Human Uniqueness). According to Haslam’s framework [21], mechanistic dehumanization occurs when people are denied the former characteristics (i.e., seen as object-like) and animalistic dehumanization occurs when they are denied the latter (i.e., seen as animal-like).

A significant proportion of research in this vein has explored the objectification of women using the tools of experimental social psychology [22–25]. Studies have shown that men (and sometimes women) perceive women as animal-like or object-like, especially as a function of the degree to which they are sexualized [26–28]. These perceptions are often assessed using measures of implicit associations [29], on the understanding that people may lack introspective awareness of what drives them.

Numerous studies have demonstrated that objectification is associated with a range of undesirable outcomes for women. For example, experiencing objectification is associated with lower relationship satisfaction [30–33]. More directly germane to the arguments of feminist theorists, however, is evidence that objectification promotes violence against women. For example, Gervais et al. [34] showed that self-reported perpetration of sexual objectification was significantly associated with the perpetration of sexual violence by heterosexual college men, Vasquez et al. [35] found that men who focused on a woman’s appearance rather than personality punished her more aggressively following a provocation, and Rudman and Mescher [36] demonstrated that men who implicitly associated women with animals or objects had relatively high propensities for sexual harassment and violence. Exposure to objectifying depictions of women on TV has also been shown to increase men’s subsequent engagement in gender-based harassment [37].

These findings relate to violence directed towards female strangers or women in general, but other research findings point to a possible role of objectification in IPV specifically. Men who objectify their female partners self-report higher rates of sexual pressure and coercion [31, 38]. Men who engage in more sexual objectification of women report higher levels of psychological and physical IPV [39], and those who sexually objectify their female partners hold more accepting attitudes toward dating violence [40]. In a study of heterosexual couples, Pizzirani and Karantzas [41] found that men’s subtle denials of their partner’s humanity were longitudinally associated with engaging in emotional abuse. Finally, Jonnson and colleagues [42] showed that men with a history of perpetration of severe physical and psychological IPV objectified women more than non-offenders.

Studies such as these indicate that objectification is implicated in violence against women, including violence that targets romantic partners. However, the body of research on objectification and IPV is relatively small and it has several limitations. First, it has not consistently assessed objectification in detail, such as by examining the characteristics that men deny to women explicitly or implicitly when objectifying them. Instead, most existing studies assess objectification as men’s chronic focus on their partner’s physical appearance or define particular behaviors as dehumanizing rather than directly evaluating how partners are perceived. Second, these studies have not consistently clarified whether it is specifically the romantic partner who is objectified or women in general. Third, specific studies have addressed a relatively narrow range of behaviors, with most individual studies focusing on sexual or psychological expressions of violence. Fourth, no existing studies of objectification and IPV have furnished experimental evidence that objectification causes IPV.

In the present research we aimed to build on the emerging body of research on the role of objectification in men’s perpetration of IPV against women, and to remedy these evidence gaps in the process. In three studies, we examined objectification in detail within a dehumanization framework, such that people can be subtly dehumanized by being implicitly associated with animals or objects [21], the latter association representing a specific, theoretically grounded model of objectification. We examined objectification of women in general and intimate partners in particular and examined several forms of violent behavior. Finally, we tested whether an experimental manipulation of objectification led to increased violence.

In Study 1 we examined whether men’s hostile sexism and tendencies to implicitly objectify or dehumanize women predicted their self-reported perpetration of psychological abuse, physical violence, or sexual coercion against a current female romantic partner. In Study 2 we replicated Study 1 but measured implicit objectification and dehumanization of individual romantic partners rather than women in general. We also supplemented the self-report measures of violence with a behavioral task that assessed aggression toward partners under conditions of imagined provocation. In Study 3 we manipulated rather than merely measured objectification, testing experimentally whether focusing on a romantic partner’s physical appearance rather than personality increases men’s tendencies to objectify and aggress against her. Together our studies aimed to clarify the psychological roots of IPV.

## Study 1

The first study addressed existing evidence gaps by examining whether implicit objectification and dehumanization predict heterosexual men’s tendencies to victimize their current female partner. We measured these implicit perceptions using a popular social cognition task that assesses nonconscious associations between women as a group and two kinds of non-human entities: animals and inanimate objects [21]. We measured victimization using self-report scales assessing IPV in sexual, physical, and psychological domains, specifically men’s attempts to sexually pressure and coerce their partners into unwanted sex, and the frequency of their perpetration of physical violence and psychological abuse. In line with previous findings [31, 36, 38], but extending them into the realm of romantic relationships, we hypothesized that stronger associations between women and objects and/or animals would be associated with higher levels of violence perpetration.

In Study 1 we also examined how hostile sexism was related to the implicit associations and to IPV. A systematic review has shown that hostile attitudes and behaviors towards women are moderately associated with men’s IPV perpetration [43], with measures of hostile sexism consistently doing so [44–46]. An older meta-analysis found that general hostility towards women was consistently associated with the likelihood of engaging in sexual violence against women [47]. Together, this research justifies the hypothesis that hostile sexist attitudes would predict the IPV measures in the present study. In view of the existing evidence that hostile sexism is associated with IPV perpetration, it is important to determine whether implicit perceptions of women have independent associations with it. Whether hostile sexism is related to these implicit perceptions of women is unclear. Some studies have found a positive association, with men who score high in hostile sexism more likely to objectify sexualized women [48] and to perceive women as having lesser humanity [49]. However, Rudman and Mescher [36] found no statistically reliable correlation between their participants’ hostile sexism and their tendencies to implicitly perceive women as animal- or object-like. Study 1 therefore explored the relationship between hostile sexist attitudes and implicit perceptions rather than hypothesizing a connection.

### Method

#### Participants

A power analysis using G*Power 3.1 [50] with an expected small effect size (*r* = .20) based on comparable previous studies [e.g., 36] suggested a sample size of 193 to achieve 80% power. Participants were recruited via Amazon Mechanical Turk (MTurk) with the requirements that they were male, heterosexual, 18 to 35 years old, and involved in a committed romantic relationship for at least one year. The sample was restricted to younger men as research indicates they perpetrate IPV at higher rates than older men [2]. Three hundred participants completed the study but 85 were removed for failing at least one exclusion criterion: failure to complete both parts of the study (*n* = 31), use of deception, for example by attempting to complete the study twice (*n* = 13), reporting they found the study language confusing or hard to follow (*n* = 6), failing at least two of the three attention check items (*n* = 5), and overly rapid response latencies (>10% of trials faster than 300ms) on the reaction-time task (*n* = 69), consistent with standard practice with implicit measures [51–53]. The final sample consisted of 215 men (*M*_age_ = 28.84 years, *SD* = 3.85, age range: 18–35), who were majority White (74%; Latino, 9%; African American, 7%; Asian, 7%; American Indian, 1%; multiracial, 3%). Forty percent of participants had been in their current relationship for over 12 months, and the rest, for over two years, and most (75%) lived with their romantic partners. More than half (58%) had completed either a 2-year college or 4-year bachelor’s degree, and 41% identified as liberal or very liberal compared to 19% as conservative or very conservative.

#### Materials

*Implicit objectification measure*. We used the Brief Implicit Association Test (B-IAT) [53] to measure automatically activated associations between target words representing men (*he*, *his*, *man*, *men*, *male*, *boy*) or women (*she*, *her*, *woman*, *women*, *female*, *girl*), matched with words characteristic of either animals (*animal*, *instinct*, *paw*, *snout*, *nature*, *hibernation*), objects (*object*, *tool*, *device*, *thing*, *instrument*, *machine*), or humans (*human*, *culture*, *logic*, *rational*, *society*, *mind*). Six neutral words were used as background stimuli (*sunset*, *yellow*, *blue*, *orange*, *green*, *dust*). These 36 words were selected based on previous implicit objectification research [28, 36].

The B-IAT is a widely used measure that examines the reaction times for classification decisions involving pairings of target categories [51, 53], such as women and animals. Quicker responses are inferred to indicate stronger cognitive associations between the focal concepts, which in theory facilitate classification. The B-IAT’s psychometric properties are well established [51, 53, 54] and similar to the popular Implicit Association Test (IAT) [55]. An advantage of the B-IAT is that it measures the absolute strength of single associations (e.g., women with animals) rather than only the strength relative to other associations (e.g., men with animals).

In the present study, the B-IAT was closely modelled on Rudman and Mescher [36] Study 2. Participants completed 10 blocks of trials. In each block, a target category (e.g., women) and a descriptor (e.g., animal)–the “focal categories”–are prominently presented at the top of the screen and a series of words is presented on the middle of the screen. Participants are asked to respond to each word as quickly as possible by pressing the right “P” key if the word matches either focal category or the left “Q” key if it does not. The first of the 10 blocks showed the general instructions and introduced the task with practice trials. Next, blocks 2–5 and 6–9 followed an identical procedure: each set began with a block of 24 practice trials, followed by three blocks of 44 critical test trials each. The only difference between these block sets was that if the first block set (i.e., blocks 2–5) corresponded to categorizing *women* with animal, object, and human constructs, the subsequent block set (i.e., blocks 6–9) would categorize *men* with the same constructs and in the same order. Block order was counterbalanced to minimize order effects. Although there were 12 possible block order combinations (i.e., 2 B-IAT targets [men, women] crossed with 6 possible B-IAT orders of the three entities [animal, object, human]), we only included the six combinations that Rudman and Mescher employed in their research. Finally, block 10 included two questions asking for participant handedness (*right/left*) and previous participation in similar tasks (*yes/no*).

We computed four separate B-IAT association scores: women-animal, women-object, men-animal, and men-object. Each score was based on two blocks of 44 test trials each–the first four prefatory trials in each block were considered practice trials and excluded from analysis [51]–using the D-statistic [55], an effect-size-like measure with a possible range of -2 to +2. For example, the women-animal B-IAT score was generated using the response latency difference between the blocks involving women-animal and women-human pairings, with D>0 if response latencies were quicker for the former pairing.

*Abuse within Intimate Relations Scale (AIRS) [56].* This scale was used to assess how often participants had engaged in violence against their romantic partner. This scale is a psychometrically sound measure of partner abuse within young adults, originally designed to identify college students at risk of perpetrating IPV. While existing IPV scales measure the perpetration of more severe forms of abuse and violence (e.g., CTS) [57], the AIRS was developed to tap into more subtle, less severe forms of abusive behavior and is considered appropriate for identifying precursors to violence among young non-clinical populations [56]. The scale has 26 items and two factors. The first factor, Psychological Abuse (α = .92), has three subscales: Emotional Abuse (7 items, *“I have mocked my partner*,*”* α = .89), Deception (4 items, *“I have lied to my partner*,*”* α = .80), and Verbal Abuse (5 items, *“I have screamed at my partner*,*”* α = .80). The second factor, Physical Violence (α = .94), has two subscales: Overt Physical Violence (7 items, *“I have physically attacked my partner*,*”* α = .92), and Restrictive Violence (3 items, *“I have grabbed my partner’s arm tightly*,*”* α = .86). The scale asks participants about frequency of perpetration for each of the 26 types of violent behavior in their current romantic relationship, using a 3-point scale (0 = *Never*, 1 = *Once*, 2 = *Twice or more*). Scores are calculated by summing responses across items, with higher scores indicating greater perpetration of IPV (scale range: 0 to 52).

*Sexual Coercion in Intimate Relationships Scale (SCIRS) [58].* The SCIRS is a 34-item scale that measures men’s self-reported use of blatant and subtle psychological and behavioral tactics of sexual coercion in romantic relationships (e.g., hints, threats, withholding of resources, and violence to obtain sex). The scale has satisfactory validity (convergent, discriminant, and predictive) [58–62]. Moreover, previous studies using the SCIRS have primarily collected data from North American heterosexual young adults [63], making it a suitable measure to use for our research purposes and population. The scale asks participants to report how often each of 34 acts have occurred in their romantic relationship, indicating frequency as *“act did not occur*,*” “act occurred 1 time*,*” “act occurred 2 times*,*” “act occurred 3 to 5 times*,*” “act occurred 6 to 10 times*,*” “act occurred 11 or more times”*. The scale has three subscales: Resource Manipulation/Violence (15 items, *“I gave my partner gifts or other benefits so that she would feel obligated to have sex with me*,*”* α = .96), Commitment Manipulation (10 items, *“I told my partner that if she were truly committed to me she would have sex with me*,*”* α = .92), and Defection Threat (9 items, *“I told my partner that other women were interested in a relationship with me*, *so that she would have sex with me*,*”* α = .96). Scores are calculated by summing responses across items within subscales, with higher scores indicating greater perpetration of sexual coercion (scale range: 0 to 170).

*Sexual Pressure Scale for Women-Revised (SPSW-R) [64].* The SPSW-R is a reliable scale originally designed to measure how much young heterosexual women feel pressured or forced to engage in unwanted sexual acts by their romantic partners. For the purpose of this study, and similarly to Ramsey and Hoyt [38], we modified the scale to reflect how much men sexually pressure women in romantic relationships to engage in sex. For example, the item *“If my partner wants sex*, *it’s my responsibility as his woman to have sex with him”* was modified to *“If I want sex*, *it’s my partner’s responsibility as my woman to have sex with me*.*”* Only two of the SPSW-R’s four subscales were included in the study: Men Expect Sex (the perception that men view sexual intercourse as a highly important aspect of the relationship, 5 items, α = .80) and Women’s Sex Roles (the sexual behaviors women are expected to follow to find and hold onto a male partner, 5 items, α = .84). For the former subscale, participants report how often each of five situations have occurred in their romantic relationship (e.g., *“I make my partner feel like she owes me something and should have sex with me”*), using a 5-point Likert-type scale ranging from 1 (*never*) to 5 (*always*). For the latter, they report how much they agree with five statements about women adhering to gender stereotypical expectations to engage in sex (e.g., *“A woman needs to please her man sexually to hold onto him”*), using a 5-point Likert-type scale ranging from 1 (*definitely do not agree*) to 5 (*definitely agree*). Scores are calculated by averaging across items in each subscale and higher scores reflect men’s greater engagement in, and agreement with, tactics to pressure their female partner into unwanted sex.

*Ambivalent Sexism Inventory (ASI) [65].* The ASI consists of two 11-item subscales that measure hostile and benevolent sexism (HS & BS). The HS subscale represents sexist antipathy towards women (e.g., *“Most women fail to appreciate fully all that men do for them*,*”* α = .92), whereas the BS subscale represents attitudes towards women that appear to be subjectively positive but patronizing (e.g., *“Women should be cherished and protected by men*,*”* α = .86). Items are rated on a 6-point Likert-type scale ranging from 1 (*strongly disagree*) to 6 (*strongly agree*), with higher scores reflecting more sexist attitudes. Only the HS subscale was used in the study. The ASI has satisfactory convergent, discriminant, and predictive validity.

*Demographics*. Participants reported information on age (self and romantic partner), gender (self and romantic partner), sexual orientation, relationship status, length of romantic relationship, living arrangements (whether the respondent cohabitated with the romantic partner [yes/no]; when couples did not cohabitate, participants were asked about the living proximity measured in time [e.g., 1 hour distance]), country of birth, ethnicity (self and romantic partner), socioeconomic status (highest level of education completed and yearly income), employment status, religion, religiosity, political stance, membership of specific groups (e.g., sporting teams), and disability (self and romantic partner).

#### Procedure

Participants accessed the survey remotely via the MTurk website by selecting to complete a task titled *Perceptions and Opinions About Romantic Relationships*. There, they followed a link to the survey and completed a screener page to verify eligibility. If eligible, participants then read the plain language statement and provided electronic informed consent. Immediately after, participants were presented with measures assessing: (a) the extent to which they implicitly associate women (and men) with objects and animals, (b) their perpetration of violence within the intimate relationship (sexual, physical, and psychological), (c) sexist attitudes towards women, and (d) demographic information about themselves and their romantic partner. The order of presentation for all experimental measures (implicit task and questionnaires), and the items within each questionnaire, were randomized for each participant. Demographic information was always presented last, followed by a debriefing statement. Participants completed the study within 25 minutes. The study was approved by the University of Melbourne’s Human Research Ethics Committee. Written consent was obtained, and data collection took place between July 1 and July 31 2016.

### Results

#### Preliminary analyses & data preparation

Following scoring protocols in IPV research [57, 66, 67], SCIRS responses that referred to frequency ranges were converted to number of sexually coercive acts by taking the midpoint of the instrument’s third and fourth response categories (i.e., *“act occurred 3 to 5 times*,*”* and *“act occurred 6 to 10 times*,*”* recoded to 4 and 8, respectively) and 16 for the *“11 or more times”* response category. The number of acts across the items in each subscale was then summed to generate a total frequency score. Inspection of correlations among AIRS, SCIRS and SPSW-R subscales found these were very high within each factor, so responses within each scale were collapsed into four parsimonious IPV measures: AIRS psychological abuse, AIRS physical abuse, SCIRS sexual coercion, and SPSW-R sexual pressure. Analyses revealed no significant relationships between demographic variables (age, ethnicity, education level, political orientation, income, relationship length) and any of these variables, so demographic information was not considered further in the analyses.

Mixed factorial ANOVAs examining the mean response latencies found no significant evidence of order effects (i.e., whether blocks of trials involving women or men, or animals, objects or humans were completed first) so data from all orders were merged. In the merged data, the women-object and men-object B-IATs yielded small D-scores (*M*_female_ = .14, *M*_male_ = .11) that did not differ from each other, *t*(214) = 0.98, *p* = .329, but were both greater than zero, female: *t*(214) = 5.35, *p* < .001, *d* = .36, and male: *t*(214) = 3.95, *p* < .001, *d* = .27. Women-animal and men-animal D-scores were also small (*M*_female_ = .03, *M*_male_ = -.02) and did not significantly differ from each other, *t*(214) = 1.59, *p* = .113, or from zero (*p*s > .05). The correlation between the women-animal and women-object B-IATs was significantly positive, *r*(215) = .34, *p* < .001, as were men-animal and men-object B-IATs, *r*(215) = .43, *p* < .001.

#### Predictors of IPV

Mean scores and distributional characteristics of the IPV measures are presented in Table 1 and the intercorrelations of the primary measures are presented in Table 2. High levels of positive skew, such as those shown in Table 1, are typical in violence research [68] and are often made amenable to statistical analysis by application of statistical transformations [e.g., 69], although these are challenged by count data with rare events. Models based on the Poisson distribution are the best-known statistical methods to analyze such data [70, 71] but our data violated its equidispersion assumption. Consequently, we used the more flexible negative binomial (NB) model [71, 72], as recommended by Swartout et al. [68].

**Table 1:** Descriptive statistics for the IPV variables, Study 1.

Variable	*M*	*SD*	Skew	Min, Max	No. of perpetrated acts of IPV			
					0	1–5	6–10	> 10
Psychological abuse	10.92	8.94	0.66	0, 32	12.1%	21.4%	20.1%	46.6%
Physical violence	1.15	3.29	3.64	0, 20	78.1%	14.4%	4.2%	3.4%
Sexual coercion	10.97	28.97	4.19	0, 204	52.6%	21.9%	4.2%	22.1%
Sexual pressure	1.82	0.79	1.05	1, 4.50	-	-	-	-

*Note*. *N* = 215, skew standard error = .166

**Table 2:** Summary of Spearman’s rank correlation coefficients for all variables, Study 1.

Scale	1	2	3	4	5	6
1. Women-animal	1					
2. Women-object	.39**	1				
3. Psychological abuse	.00	.13	1			
4. Physical violence	.00	.14*	.39**	1		
5. Sexual coercion	.11	.14*	.30**	.43**	1	
6. Sexual pressure	.00	.10	.35**	.34**	.55**	1
7. Hostile sexism	.11	.08	.23**	.20**	.45**	.55**

*Note*. *N* = 215. *p < .05.

**p < .01.

To test whether implicitly associating women with animals or objects predicted self-reported perpetration of psychological abuse, physical violence, and sexual coercion within the romantic relationship, we ran a NB regression using the women-animal, women-object, and HS scores as predictors for each of the IPV outcomes. To facilitate the interpretation of the NB regression coefficients in terms of incidence rate ratios (IRR; i.e., the exponentiated coefficients), prior to the analyses the raw values for the independent variables were standardized using a z-score transformation (*M* = 0, *SD* = 1) so that the measures were on the same metric (i.e., standard deviations). The IRR represents the percentage change, increase or decrease, in the observed counts and quantifies the direction and strength of the relationship between the predictor and outcome [72, 73]. There was no evidence of systematic patterns of outliers or of extremely influential cases, assessed by visually inspecting plots of mean predicted values against deviance residuals and by Cook’s distance values >1 [74].

Findings of the NB regression analyses are summarized in Table 3. The full regression model for psychological abuse showed a statistically significant improvement in fit over the null model, likelihood ratio (LR) χ^2^ (3, *n* = 215) = 7.80, *p* = .050, but only HS had a significant effect. The regression model for physical violence was also significant, LR χ^2^ (3, *n* = 215) = 12.03, *p* = .007, and both women-object associations and HS were significant predictors, a standard deviation unit increase in the predictor variables increased the rate of violence by 93% (IRR = 1.93, 95% CI: 1.15, 3.25) and 92% (IRR = 1.92, 95% CI: 1.20, 3.06), respectively. The regression model for sexual coercion was again significant, LR χ^2^ (3, *n* = 215) = 32.12, *p* < .001. Both the women-object associations and HS were associated with an increased likelihood of perpetrating sexual coercion, where a standard deviation unit increase in the predictor variables increased the rate of sexual coercion by 40% (IRR = 1.40, 95% CI: 1.01, 1.95) and 334% (IRR = 4.34, 95% CI: 2.68, 7.04), respectively. Because sexual pressure was not a frequency count variable, a multiple linear rather than NB regression analysis was conducted and was significant, *F*(3, 211) = 24.15, *p* < .001, adj. *R*^*2*^ = .25. Only HS significantly contributed to the model (β = 0.50, *p* < .001), with women-object (β = 0.09, *p* = .139) and women-animal associations (β = -0.08, *p* = .204) nonsignificant.

**Table 3:** Summary of negative binomial regression analyses for the variables predicting IPV, Study 1.

Variable	*b*	SE	IRR	Wald *χ*^*2*^	*df*	*p*
	Psychological abuse					
Intercept	2.38	.07	10.73	1292.31	1	< .001
Women-animal	-.02	.08	.98	.07	1	.793
Women-object	.11	.07	1.11	2.01	1	.156
Hostile sexism	.16	.07	1.17	5.34	1	.021
	Physical violence					
Intercept	-.16	.22	.85	.53	1	.496
Women-animal	-.19	.33	.83	.32	1	.570
Women-object	.66	.27	1.93	6.09	1	.014
Hostile sexism	.65	.24	1.92	7.43	1	.006
	Sexual coercion					
Intercept	1.93	.16	6.92	141.93	1	< .001
Women-animal	-.08	.21	.92	.16	1	.694
Women-object	.34	.17	1.40	3.98	1	.046
Hostile sexism	1.47	.25	4.34	35.44	1	< .001

*Note*. *N* = 215. *b* = Unstandardized coefficient; SE = Standard error; IRR = Incidence-rate ratio; *df* = degrees of freedom.

### Discussion

Study 1 demonstrated that implicit views of women are associated with men’s tendencies to engage in some forms of IPV. In particular, implicit associations between women and objects predicted self-reported tendencies to perpetrate physical violence and sexual coercion with a romantic partner. These findings are in accord with previous work on the role of objectification- and dehumanization-related processes in sexual coercion and violence in particular [31, 36, 38]. Implicit objectification, the nonconscious association of women with inert objects or instruments, appears to be implicated in the psychology of IPV, consistent with arguments from feminist thinkers and our hypothesis. In theory, this association might be linked to violence by undermining emotional concern for women, viewing them instrumentally or as possessions, or seeing them as fungible [21], but the present study cannot clarify the precise mechanisms. As a correlational study it also cannot make any claim that implicit objectification causally contributes to men’s IPV.

The connection between men’s objectification of women and their perpetration of violence in romantic relationships is a novel finding, although others have found evidence consistent with dehumanization of partners within intimate relationships being associated with a range of undesirable and abusive behaviors [41]. Less novel is the consistent association between HS and the violence indicators, which accords with substantial previous research [44, 46]. However, the fact that implicit associations of women with objects predicted some forms of violence independently of this well-established predictor, while being uncorrelated with it, reinforces the importance of the former finding. In contrast, the consistent lack of evidence that implicit associations between women and animals predicted men’s IPV was unexpected and conflicts with some previous research [36]. This pattern of null effects suggests that woman-object associations are more critical to violence in the specific and previously unstudied context of intimate relationships than woman-animal associations. It remains possible that animalistic perceptions of women are implicated in forms of aggressive behavior towards women outside romantic relationships, such as in sexual harassment or other kinds of aggression towards female strangers.

Although romantic relationships may be a distinctive context for gendered violence, marked by intimacy and interdependence, our findings indicate that violent behavior is not uncommon within them. In this study, almost one quarter admitted to perpetrating at least one act of physical violence against their current partner, close to 50% at least one act of sexual violence, and almost 90% some form of psychological abuse. These rates are comparable to those found globally [1] and in other empirical studies of IPV prevalence [75–79]. As participants may have had strong motivations to under-report violent behavior, these rates are troubling and highlight how IPV is rife among young adults.

Although we found promising evidence of a link between implicit objectification and men’s perpetration of IPV, Study 1 has limitations. First, by focusing on implicit views of women as a general class it is likely to miss important components of objectification that are involved in romantic relationships. In particular, men’s objectification of their partners might be expected to be at least equally related to their behavior towards them. Second, self-report measures of violence perpetration raise validity concerns including social desirability bias and retrospective memory distortions. Ethically evaluating violent behavior in a more behavioral manner would improve ecological realism. In Study 2 we sought to replicate Study 1 and address these two limitations, assessing men’s implicit associations with their female romantic partners and employing a proxy behavioral measure of violence.

## Study 2

Study 1’s findings indicate that men’s objectification of women is associated with their perpetration of violence in romantic relationships, assessed as their self-reported rates of sexual and physical violence. These findings offer preliminary support for the role of objectification in IPV. However, two further questions arise. First, does objectification of intimate partners as individuals rather than women as a class also contribute to violence, and second, can tendencies towards violent behavior be assessed in a more ecologically realistic fashion than by self-report questionnaires. In Study 2 we extended Study 1 in an attempt to address these questions.

Whether objectification of romantic partners might be implicated in IPV is uncertain. On the one hand, the closeness of romantic relationships and the high degree of interdependence that characterizes them could protect women from being objectified, or at least from being objectified in destructive ways. Nussbaum [12] posits that the context of human relationships matters when it comes to objectifying practices, and that romantic relationships are a place where objectification can be benign or even positive. On the other hand, romantic relationships are not free of conflict, inequality, and disrespect. An emerging literature indicates that forms of objectification and subtle dehumanization occur in intimate relationships and can have harmful consequences.

Qualitative studies outside psychology indicate that viewing a close partner in objectifying ways, as a thing to be owned and controlled, plays a role in men’s use of violence within intimate relationships [80–83]. Within psychology, a study of heterosexual college males found that those who reported frequently judging their romantic partner’s body were more likely to use tactics to pressure and coerce them into having unwanted sex [38]. Pizzirani and Karantzas [41] found tendencies to dehumanize intimate partners, such as seeing them as cold and unfeeling, was associated with perpetration of emotional and physical abuse. Partner-objectification has also been shown to predict lower levels of relationship satisfaction [33]. The existence and impact of objectification in close relationship is therefore increasingly plausible.

In addition to examining objectification of romantic partners rather than women in general, Study 2 uses a behavioral task as a more direct, laboratory-analogue measure of physical aggression to complement the self-measures retained from Study 1. The task involved performing imaginary acts of violence against a visual representation of the current romantic partner. Although not a direct measure of actual aggression perpetration, this behavioral proxy task, described in the Method section, is more proximal to actual aggression than self-reports and is not undermined by possible memory and other biases. We predicted that men who score high on implicit partner-objectification IAT would show greater aggression on this task. As with Study 1, HS was included in the study as an additional, known predictor of IPV [43, 44, 46, 47, 69], and was also expected to predict aggressive behavior.

### Method

#### Participants

As in Study 1, we recruited USA-based participants from MTurk. Three hundred and ninety-four men who had not taken part in Study 1 were sampled with the same inclusion criteria. We excluded a total of 69 participants for a variety of reasons (some failed more than one): failing to complete both parts of the study (*n* = 5); use of deception (*n* = 13); finding the language of the study confusing or hard to follow (*n* = 1); failing at least one of the three attention checks (*n* = 1); excessively fast IAT responses (*n* = 63); and deciding to withdraw their data on completion of the survey (*n* = 11). The final sample contained 325 men (*M*_age_ = 29.75 years, *SD* = 3.71, age range: 20–35), who were majority White (82%; Latino, 6%; African American, 6%; Asian, 6%; and American Indians or multiracial, <1%). Forty-two percent of participants had been in the current relationship for over 12 months, and the rest, for over two years. Most (72%) lived with their romantic partners, had completed either a 2-year college or 4-year bachelor’s degree (53%), and identified as liberal or very liberal (52%).

#### Materials

*Implicit partner objectification measure*. A relational Single-Category IAT (SC-IAT) [84] was used to assess the extent to which men implicitly associated their female romantic partner with animals or objects. The SC-IAT is a modified version of the IAT that uses only *one* target category instead of the *two* target categories required for the original task. It can be used to evaluate automatically activated associations for concepts without an obvious contrast, as when assessing perceptions of intimate partners. In the present research the SC-IAT was used to evaluate implicit associations between words representing the target concept “Romantic Partner” (*partner*, *lover*, *significant other*, *beloved*, *loved one*, *companion*) and the same descriptor words characteristic of animals, objects, or humans employed in Study 1, based on prior research on objectification [28, 36, 85].

The SC-IAT measures the absolute strength of the non-relative associations between the single target concept and the matched dimensions using participants’ reaction times [84, 86–88]. During the task, the single target concept (e.g., romantic partner) is paired with a descriptor category (e.g., human) and prominently presented on one top corner of the screen. On the opposite top corner, the second descriptor category is presented (e.g., object). A word stimulus is then displayed in the middle of the screen. Participants must rapidly classify each stimulus as belonging to the right or left category. Classification should be facilitated when the respondents’ cognitive associations between the concepts presented match a pairing of target-descriptor categories. The SC-IAT has been shown to have adequate psychometric properties including internal consistency, test-retest reliability, convergent and discriminant validity, and predictive validity [84, 89, 90].

In the present study, participants completed two SC-IATs consecutively, one assessing associations between romantic partner and objects and the other between the partner and animals. Each SC-IAT contained five blocks of trials: one general practice block, a short (24-trial) practice block for one target-descriptor pair (e.g., partner or object), a longer (72-trial) block assessing that pairing, a short practice block for the other pair (e.g., partner or human), and a longer block assessing this pairing. On completion, participants were presented with a final block that asked them about handedness (*right/left*) and previous participation in similar tasks (*yes/no*). Order of presentation for the two SC-IATs and for the blocks within each SC-IAT was counterbalanced between participants to minimize order effects. SC-IAT scores were based on the longer blocks of 72 test trials (blocks 3 and 5), with all practice trials excluded from analysis [84]. Then, the SC-IAT effect (or D-score) was calculated using the improved scoring algorithm described by Greenwald and colleagues [55]. Positive D values (> 0) were computed to reflect quicker latencies to pairing the romantic partner with animals and/or objects than with humans.

*Self-report measures*. We retained three self-report measures of violent tendencies and sexism from Study 1. The 26-item AIRS [56] was used to assess how often participants engaged in psychological and physical violence in their romantic relationship (Psychological Abuse factor [α = .90], Physical Violence factor [α = .90]). The 34-item SCIRS [58] assessed how often participants had perpetrated sexual violence against their female partner (Resource Manipulation/Violence [15 items, α = .93], Commitment Manipulation [10 items, α = .91], and Defection Threat [9 items, α = .96]). The scale rated the frequency of perpetration for each of the 34 sexually violent behaviors in the last six months of the current relationship. The ASI [67] was again used to measure sexist attitudes towards women, although the 12-item short-form version was administered in Study 2. This form, which has good psychometric properties [91], has two six-item subscales assessing HS (α = .92), and BS (α = .85). Participants also completed the same demographic questionnaire as in Study 1.

*Proxy measure of violent behavior*. In addition to the above self-report questionnaires, Study 2 used the Voodoo Doll Task (VDT) [92] as a behavioral proxy for physical IPV. The task gives participants the opportunity to intentionally harm a computer-based image of a doll representing the romantic partner by stabbing it with pins. DeWall and colleagues [92] posit that harming the representation of the person (i.e., the doll) has important psychological similarities to the process of causing actual harm to the person that the doll represents. Although most people would not endorse the belief that stabbing a doll with pins actually harms the person it represents, willingness to do so may imply a lack of inhibition of aggressive behavior towards the person. Several studies indicate that the VDT is a valid and reliable test for the assessment of physical aggression in the context of romantic relationships [92–94], and it is widely used in the anger and aggression literatures, including in work on IPV [95, 96].

The VDT was employed in conjunction with a provocation manipulation, in which participants read about a scenario in which their partner behaved in a way that might provoke jealousy and a sense of being disrespected. The scenario described the couple out at a bar, and while the participant was getting drinks, another man would start flirting with his female partner. During this interaction, she would express clear romantic interest in the new suitor (e.g., engaging in flirtatious physical contact) and discontent about her current relationship (e.g., making statements such as *“things just aren’t the same anymore”* and *“I really don’t know why I’m still dating him”)*. To ensure participants read the vignette carefully and immersed themselves in the situation being described, the ability to continue advancing throughout the survey was disabled for 2 minutes. This manipulation was employed because real or perceived interpersonal provocation promotes aggression [95, 97] and is a situational predictor of IPV [85, 92, 96, 98]. Provocations such as jealousy, humiliation, and rejection are commonly cited as primary precipitating factors for both non-lethal [79, 99–101] and lethal IPV [100, 101].

*Emotion rating task*. Following the provocation, participants rated themselves on a scale from 1 (not at all) to 9 (extremely) on eight emotions in response to the question “Thinking about your girlfriend’s behaviour in the scenario, please read each item and indicate to what extent you feel this way right now, that is, at the present moment.” The emotion terms–provoked, disrespected, hostile, angry, humiliated, happy, appreciated, and supported–were selected to assess anger and upset in response to the provocation. The scale was computed as the mean rating following reverse scoring of the last three emotion terms and had good internal consistency (Cronbach’s α = .81).

#### Procedure

Participants accessed the survey via the MTurk website and if found to be eligible read the plain language statement and provided electronic informed consent. They then proceeded to complete the SC-IAT, after which they provided the name or nickname of their romantic partner to enable personalization of the partner-provocation vignette and the VDT. Participants then completed in individually randomized order the self-report measures of violence perpetration, referencing their current relationship and the previous six months, and of sexism. They then read the immersive provocation vignette and completed the emotion rating task. Participants then completed the VDT, which offered the participants the opportunity to stab with pins a doll representing their romantic partner–identified by name in the vignette and the VDT instructions–for a period of 2 minutes. Finally, they completed the demographic questionnaire as in Study 1 and read the debriefing statement. The study was approved by the University of Melbourne’s Human Research Ethics Committee. Written consent was obtained, and data collection took place between January 1 and January 31 2018.

### Results

#### Preliminary analysis & data preparation

A mixed factorial ANOVA conducted on the SC-IAT mean response latencies found no order effects and the data were collapsed across orders. D-scores for the partner-object SC-IAT were small (*M* = -.16) significantly below zero, *t*(324) = -9.97, *p* < .001, *d* = .55, implying that partners were associated with human words more than object words on average. D-scores for the partner-animal SC-IAT were also small (*M* = -.13) and negative, *t*(324) = -8.46, *p* < .001, *d* = .47. Partner-object and partner-animal SC-IATs correlated positively, *r*(325) = .16, *p* = .003. As in Study 1, SCIRS scores were recalculated to yield frequencies of sexually coercive acts [57, 66, 67], and SCIRS and AIRS subscales were found to be highly correlated and so combined into separate physical violence and psychological abuse (AIRS) and sexual coercion (SCIRS) factors. Also as in Study 1, no demographic variable was significantly associated with any IPV measure, so they were not included in the primary analyses. Finally, analysis of the post-provocation vignette feelings ratings indicated that participants were emotionally engaged by it (*M* = 7.50, *SD* = 1.23; on a scale from 1 = *not at all* to 9 = *extremely*).

#### Descriptive statistics and correlations

Mean scores and distributional characteristics of the IPV measures are presented in Table 4 and the intercorrelations of the primary measures are presented in Table 5. Consistent with Study 1 and as expected with violence frequency data, all the outcome variables were non-normally distributed as assessed by the Shapiro-Wilk’s test (*p*s < .05) and highly positively skewed. Table 5 indicates that unlike Study 1, partner-animal and partner-object SC-IATs had no positive correlations with any of the IPV variables, were positively correlated, and the former was negatively correlated with psychological abuse. As expected, HS was strongly correlated with all the IPV measures.

**Table 4:** Descriptive statistics for the IPV variables, Study 2.

Variable	*M*	*SD*	Skew	Min, Max	No. of perpetrated acts of IPV			
					0	1–5	6–10	> 10
Psychological abuse	11.04	8.66	0.61	0, 32	10.2%	24.0%	20.4%	45.3%
Physical violence	0.86	2.65	4.55	0, 20	81.2%	13.2%	3.6%	1.8%
Sexual coercion	11.32	34.23	5.99	0, 336	52.3%	19.1%	8.3%	20.3%
Voodoo doll pins	3.21	9.25	6.44	0, 100	47.7%	38.5%	8.3%	5.5%
Hostile sexism	2.87	1.31	0.30	1, 6				

*Note*. *N* = 325, skew standard error = .135.

**Table 5:** Summary of Spearman’s rank correlation coefficients for all variables, Study 2.

Scale	1	2	3	4	5	6
1. Partner-animal	1					
2. Partner-object	.16**	1				
3. Psychological abuse	-.11*	.02	1			
4. Physical violence	-.07	.06	.41**	1		
5. Sexual coercion	-.04	.04	.46**	.36**	1	
6. Voodoo doll pins	-.01	.08	.30**	.19**	.34**	1
7. Hostile sexism	-.15**	-.00	.21**	.16**	.30**	.30**

*Note*. *N* = 325. *p < .05.

**p < .01.

Again, NB models were used in multivariate analyses to predict the non-normal, highly skewed, over-dispersed, and count-based outcome measures [68, 72]. To facilitate the interpretation of the NB regression coefficients in terms of IRRs, the raw values for the independent variables were again standardized using a z-score transformation (*M* = 0, *SD* = 1) so that the measures were on the same metric. The IRR represents the percentage change in the observed counts and quantifies the direction and strength of relationship between the predictor and outcome [73]. Analyses are summarized in Table 6. The full regression models predicting psychological abuse (LR χ^2^ (3, *n* = 325) = 11.69, *p* = .009), physical violence (LR χ^2^ (3, *n* = 325) = 14.59, *p* = .002), sexual coercion (LR χ^2^ (3, *n* = 325) = 18.80, *p* < .001), and the number of pins used in the VDT (LR χ^2^ (3, *n* = 325) = 23.36, *p* < .001) all demonstrated statistically significant improvement in fit over the null model. Hostile sexism was a significant predictor of all four variables, automatic partner-object associations predicted physical violence only, and partner-animal associations were unrelated to any IPV measure.

**Table 6:** Summary of negative binomial regression analyses for the variables predicting IPV, Study 2.

Variable	*b*	SE	IRR	*Wald χ* ^*2*^	*df*	*p*
	Psychological abuse					
Intercept	2.39	.05	10.87	2217.29	1	< .001
Partner-animal	.08	.05	1.08	2.16	1	.142
Partner-object	-.02	.06	0.98	.12	1	.730
Hostile sexism	.15	.05	1.17	8.05	1	.005
	Physical violence					
Intercept	-.42	.19	.66	5.06	1	.025
Partner-animal	.28	.22	1.33	1.69	1	.194
Partner-object	.46	.20	1.59	5.49	1	.019
Hostile sexism	.36	.17	1.44	4.43	1	.035
	Sexual coercion					
Intercept	2.24	.14	9.38	260.23	1	< .001
Partner-animal	.11	.16	1.12	.48	1	.490
Partner-object	.17	.16	1.19	1.21	1	.271
Hostile sexism	.57	.14	1.77	16.48	1	< .001
	Voodoo Doll Task					
Intercept	1.02	.11	2.78	88.88	1	< .001
Partner-animal	-.01	.14	.99	.01	1	.933
Partner-object	.05	.12	1.05	.21	1	.650
HS	.45	.10	1.57	19.30	1	< .001

*Note*. *N* = 325. *b* = Unstandardized coefficient; SE = Standard error; IRR = Incidence-rate ratio; *df* = degrees of freedom.

To follow up the lack of evidence for effects of implicit associations on the VDT, we repeated the NB analysis while adding three additional predictors: the level of provocation based on the emotion rating scale and terms representing the interactions between provocation level and the partner-object and partner-animal associations (products of the respective standardized variables). This analysis asks whether violent behavior is greater if the participant felt more provoked and/or if implicit objectification or dehumanization might only have effects at high levels of provocation. In the first model tested, only the provocation variable was added, and in the second model the interaction terms were added. The two models are presented in Table 7. When provocation was added to the basic model from Table 6, it was found to predict VDT responses, with greater emotional response associated with an increased likelihood of inserting pins. When we added the interaction terms in Model 2, the main effect of provocation and both new terms were statistically significant. At high levels of provocation, participants who associated their partners more with animals and objects inserted more pins into dolls representing those partners than those with less objectifying or dehumanizing associations. Adding the three predictors significantly improved model fit over the basic model presented, LR χ^2^ (6, *n* = 325) = 34.62, *p* < .001.

**Table 7:** Summary of negative binomial regression analyses for the Voodoo Doll Task including provocation, Study 2.

Variable	*b*	SE	IRR	*Wald χ* ^*2*^	*df*	*p*
Model 1						
Intercept	0.97	.11	2.71	85.54	1	< .001
Partner-animal	-.03	.14	0.97	0.06	1	.803
Partner-object	.01	.11	1.01	0.01	1	.908
Hostile sexism	.40	.10	1.49	14.79	1	< .001
Provocation	.26	.12	1.30	4.82	1	.028
Model 2						
Intercept	0.95	.11	2.59	78.74	1	< .001
Partner-animal	-.02	.13	0.98	0.03	1	.876
Partner-object	.02	.11	1.03	0.05	1	.828
Hostile sexism	.39	.11	1.47	13.42	1	< .001
Provocation	.29	.12	1.34	5.74	1	.017
Provocation X partner-animal	.27	.12	1.31	4.90	1	.027
Provocation X partner-object	.24	.12	1.28	3.98	1	.046

*Note*. *N* = 325. *b* = Unstandardized coefficient; SE = Standard error; IRR = Incidence-rate ratio; *df* = degrees of freedom.

### Discussion

The findings of Study 2 with regard to the self-report measures of violence are broadly comparable with Study 1. Hostile sexism was the dominant predictor, significantly associated with the psychological abuse, physical violence, and sexual coercion measures as it had been in the earlier study. This finding adds to substantial evidence that HS is associated with aggressive and coercive interactions towards romantic partners [102, 103], and greater perpetration of physical, verbal, and sexual IPV [44–46, 104, 105].

In direct contrast, implicit partner-animal associations assessed by the SC-IAT were unrelated to any of the measures. However, partner-object associations–a measure of partner objectification distinct from the more typical sexualization definition–was associated with the physical violence measure, although the association with sexual coercion obtained in Study 1 did not replicate. The link between implicit objectification and violence did not extend in a straightforward manner to the VDT, which was again predicted by HS. However, the post hoc finding that these implicit associations predicted VDT aggression in interaction with experienced level of provocation suggests they may be implicated in IPV in a facilitative or disinhibitory way. Seeing one’s partner as object-like may, within Finkel’s [106] I^3^ model of IPV, impel instrumentally aggressive acts and/or reduce inhibitions against such acts.

Although associations between implicit objectification and IPV were not obtained consistently or were conditional on provocation intensity, they are theoretically important in demonstrating that nonconscious perceptions of romantic partners (rather than just women in general) are involved in tendencies to act violently in relationships, independently of hostile attitudes to women. IPV appears to be rooted partly in psychological factors beyond gender-related prejudices, although experimental or longitudinal research would be required to test whether implicit objectification plays a truly causal role. Although these factors may relate to a specific relationship partner, they are unlikely to be individualized and more likely to reflect gendered cultural norms to do with the sexualization of women. Clinical psychological and qualitative studies examining implicit theories in the narratives of male perpetrators of violence against women [81, 107–110] often identify objectifying elements, such as viewing women as objects or possessions.

The results from Study 1 and 2 provide a range of preliminary findings that shed light upon the role of implicit objectification as a precursor to, or enabler of, IPV. In Study 3 we extend our focus of investigation to examine partner-objectification explicitly and directly. In search of evidence for a more direct, causal link between objectification and enacted aggression towards a romantic partner, we carried out an experimental manipulation of objectification and observed its effect on the VDT.

## Study 3

Study 1 and 2 suggest that men’s implicit objectification of women in general or their romantic partner in particular plays a role in IPV, perhaps especially when men feel provoked by their partner’s behavior. In Study 3 we extended this research by shifting attention to explicit forms of objectification and by manipulating rather than only measuring objectification. Objectification can occur at both the conscious and implicit levels [111] and to understand its role in IPV it is important to examine both levels. The study tests whether inducing objectification by having men focus on their partner’s appearance diminishes their attributions of personhood to their partner and increases proxy aggressive behavior toward her on the VDT. We again used the provocation employed in Study 2 to investigate whether situational provocation exacerbates aggression and does so especially for high objectifiers.

Manipulating objectification and observing its impacts has been a popular methodology in psychological research [e.g., 14–16, 18, 19, 112], although it has not previously been used in the context of romantic relationships. Objectification is typically induced by instructing participants to pay attention to a target’s appearance rather than their psychological qualities, based on the longstanding feminist notion that women are valued primarily on the basis of their physical body [9, 11, 113]. In research of this kind, objectification is manipulated via appearance focus but usually conceptualized as the failure to acknowledge a target’s personhood (e.g., agency, subjectivity, autonomy), an understanding that draws a close connection between objectification and dehumanization [e.g., 14, 19, 114]. Research has shown that presenting targets in sexualized or body-focused ways is associated with ascribing them with lesser humanness, mental capacity, and the moral standing that typically accompanies being human and having a mind [14, 19, 28]. However, research has yet to examine whether these perceptions apply in the context of romantic relationships.

To assess partner-objectification explicitly, we employed Haslam’s [21] humanness model and the mind perception framework [115, 116]. In the former, two dimensions of humanness are recognized: Human Nature (HN), which distinguishes people from inanimate objects, and Human Uniqueness (HU), which distinguishes them from animals. Perceiving people as lacking the respective forms of humanness amounts to mechanistic dehumanization (akin to objectification) and animalistic dehumanization, respectively. The mind perception framework defines Agency (the capacity for complex cognition) and Experience (the capacity for feeling or sentience) as separate dimensions of mind. It also invokes Moral Agency and Moral Patiency as dimensions of moral status linked to the possession of these kind of mind: people with agentic capacities are able to act morally or immorally, and people with experiential capacities can have moral or immoral things done to them. The extent to which partners deny attributes composing these dimensions to their partners represents how much they see them as lesser persons, with denials of HN, Experience, and Moral Patiency especially relevant to objectification. There is now extensive evidence that objectification manipulations bring about these perceptions. For example, objectified people are deemed to suffer less [19] and to have less moral agency [18]. As these kinds of mind denial are linked to perpetration of aggression [35–37, 117], it is important to examine them in the context of romantic relationships.

In Study 3 we therefore tested three hypotheses. First, we predicted that an objectification manipulation would increase aggressive behavior toward men’s romantic partners. Second, we predicted that this manipulation would also promote objectifying or dehumanizing perceptions of them, as it has been found to do in studies of non-intimate female targets. Third, we predicted that an interpersonal provocation would increase aggressive behavior and do so especially when romantic partners have been objectified.

### Method

#### Participants

A power analysis conducted using G*Power 3.1 [50], anticipating a small effect size in a 2 x 2 between-subjects factorial experiment, indicated a sample size of 195. A total of 250 USA-based men who had not participated in Studies 1 or 2 were recruited from MTurk, with inclusion criteria requiring them to identify as being male, heterosexual, 18 to 35 years old, and currently involved in a committed romantic relationship for at least one year. After we excluded 61 participants– 26 failed to complete the study, 13 did not follow instructions for the objectification manipulation, 11 engaged in deception to enter the study, 6 withdrew their data, 3 failed at least one reading comprehension checks, and 2 found the language of the study confusing or hard to follow– 189 participants remained (*M*_age_ = 28.62 years, *SD* = 3.86, age range: 20–35). Most identified as White (71%; African American, 8%; Asian, 8%; Latino, 7%; multiracial, 5%; 1% preferred not to answer). Forty-five percent of participants had been in their current relationship for over 12 months, and the rest for over two years, and most (68%) lived with their romantic partner and had completed either a 2-year college or 4-year bachelor’s degree. Participants reporting liberal or very liberal political orientation outnumbered those reporting conservative or very conservative views, 50% to 27%.

#### Materials

*Voodoo Doll Task*. Participants completed the same task as in Study 2 with a similar provocation manipulation, based on vignettes used in work by Slotter and colleagues [85]. Participants either read the Study 2 provocation text, in which their partner responds favorably to another man flirting with her at a bar or, in the no provocation condition, she clearly and politely refused the other man’s advances. To ensure participants immersed themselves in the situations being described, their ability to continue advancing throughout the survey was disabled for 2 minutes. After reading the text, all participants completed the same 8-item affect scale from Study 2.

*Objectification manipulation*. To induce objectification, we used a previously validated mindset manipulation task that varied the amount of focus placed on the target’s physical appearance or personality [14, 15]. In the objectification condition, participants wrote a minimum of six sentences about their romantic partner including both positive and negative features, focusing on their appearance (*“Please take some time to write your thoughts and feelings about your romantic partner’s physical appearance”)*. The no objectification condition was identical except “*partner’s personality*” replaced “*partner’s physical appearance*”.

*Humanness ratings*. To assess subtle dehumanization, participants rated 40 traits from Bastian and Haslam [118]. These included 10 Human Nature (HN) traits (five positive: *active*, *curious*, *friendly*, *helpful*, *fun-loving*; five negative: *impatient*, *impulsive*, *jealous*, *nervous*, *shy*), 10 Human Uniqueness (HU) traits (five positive: *broadminded*, *conscientious*, *humble*, *polite*, *thorough*; five negative: *disorganized*, *hard-hearted*, *ignorant*, *rude*, *stingy*), and 20 filler traits, which were not included in the analysis. The HN and HU traits have been previously validated as rating highly and distinctively on each dimension of humanness [119]. Using 7-point scales, participants rated the 40 traits four times in randomized order: (a) how much their partner possessed the traits when compared to the average woman; (b) how much the traits were part of human nature; (c) how much the traits were unique to humans rather than shared with animals; and (d) how much the traits were positive or desirable to possess (i.e., valence rating; 1 = *extremely undesirable* to 7 = *extremely desirable*). HN and HU indices to assess subtle dehumanization of the partner were created by computing within-participant partial correlations between partner ratings on the 20 traits of interest and the HN and HU ratings, respectively, controlling for trait valence ratings to ensure that (de)humanization was not contaminated by (dis)like. The two indices ranged from -1 to +1, with lower scores indicating greater dehumanization. Similar approaches to developing humanness indices have been employed in past research [14, 20, 120].

*Mind attribution*. We used the Mental State Attribution task [121] was used to evaluate participants’ ratings on their partner’s mental capabilities. The MSA is a previously validated measure of mind attribution often used in objectification research [e.g., 18, 19] that asks participants to rate the extent to which their partner possessed 20 mental states involving thoughts (5 items; e.g., *thinking*), intentions (5 items; e.g., *plans*), perceptions (5 items; e.g., *seeing*), and emotions (5 items; e.g., *fear*) on a 7-point scale (1 = *definitely does not possess*; 7 = *definitely does possess*). These items capture the two dimensions of mind perception: agency (thoughts, intentions) and experience (perceptions, emotions). Two scores were then generated by collapsing across the 10 agency items (α = .93) and the 10 experience items (α = .92). Lower scores indicated greater dementalization of the partner.

*Moral status*. A 13-item scale adapted from work by K. Gray and Wegner [116] and H.M. Gray and others [115] by Holland and Haslam [18, 112] was used to examine whether moral status was denied to participants’ partners. Five items assessed their perceived moral agency (e.g., “*In general*, *how intentional do you believe your girlfriend’s behavior is*?*”*), six items moral patiency (e.g., *“How bad would you feel if you manipulated your girlfriend*?*”*), and two items moral blame and praise (e.g., “*How much should your girlfriend be blamed for doing something bad*?”). Items were rated on a 7-point scale. The moral agency subscale, which included items to do with agency, blame, and praise had modest reliability (α = .66), so two items with low (< .30) item-total correlations were removed, yielding a small rise in reliability (α = .72). The moral patiency subscale was more reliable (α = .84). Lower scores in either subscale indicate lower levels of ascribed moral status to the partner.

*Emotion rating task*. Following the provocation manipulation, participants completed the same 8-item emotion rating task as in Study 2, with the resulting scale being highly reliable (α = .96).

#### Procedure

Participants accessed the survey via the MTurk website and followed a link to the survey and completed the eligibility screening page. If eligible, participants read the plain language statement and provided electronic informed consent. Afterwards, they read the general instructions that asked them to base all responses on their current romantic relationship and to provide the name (or nickname) of their partner. Participants were then randomized to the provocation or no provocation condition, competed the affect ratings, and then were randomized to the objectification or no objectification condition. Following this mindset manipulation, they completed the humanness, mind, and moral status measures, followed by the VDT, the demographics questionnaire, and finally a debriefing statement. The study was approved by the University of Melbourne’s Human Research Ethics Committee. Written consent was obtained, and data collection took place between March 1 and March 31 2018.

### Results

Analysis of the emotion ratings indicated that participants in the provocation condition (*M* = 7.65, SD = 1.20) had substantially more negative emotional responses than those in the no provocation condition, (*M* = 3.77, SD = 2.17), Welch’s *t*(146.95) = 15.23, *p* < .001, *d* = 2.21. To evaluate the efficacy of the objectification manipulation, two independent coders rated the focus of participants’ written evaluations of their romantic partner from 1 (*completely appearance*) to 7 (*completely personality*). Inter-rater reliability was high (Krippendorff’s α = .89) and coder ratings were therefore averaged. This averaged rating showed large differences between the no objectification (*M* = 1.89, SD = 1.01) and objectification conditions (*M* = 5.88, SD = 1.24), Welch’s *t*(184.88) = 24.34, *p* < .001, *d* = 3.53.

A series of 2 (provocation, no provocation) x 2 (objectification, no objectification) between-subjects factorial ANOVAs were conducted on the outcome variables (HN, HU, agency, experience, moral agency, moral patiency, and VDT). Descriptive statistics are shown in Table 8.

**Table 8:** Mean, standard deviation, and sample size for the variables as a function of condition, experiment 3.

	No Provocation			Provocation		
Variable	*M*	*SD*	*N*	*M*	*SD*	*N*
HN						
No objectification	0.18	0.24	43	0.16	0.22	37
Objectification	0.07	0.31	47	0.10	0.27	42
HU						
No objectification	0.08	0.27	45	0.04	0.21	42
Objectification	0.01	0.31	47	0.10	0.24	48
Agency						
No objectification	5.97	0.98	46	5.55	1.12	44
Objectification	5.64	1.13	49	5.88	1.10	50
Experience						
No objectification	5.89	1.11	46	5.53	1.26	44
Objectification	5.42	1.09	49	5.82	1.10	50
Moral agency						
No objectification	5.80	0.78	46	5.75	0.82	44
Objectification	5.84	0.70	49	5.93	0.88	50
Moral patiency						
No objectification	6.63	0.63	46	6.42	0.75	44
Objectification	6.44	0.75	49	6.45	0.72	50
Voodoo doll task						
No objectification	0.57	0.81	46	0.98	1.47	44
Objectification	0.96	2.12	49	1.70	2.70	50

*Note*. HN = Human nature; HU = Human uniqueness. Differences in sample size for the humanness variables is a result of cases where the partial within-subject correlation could not be computed due to participants showing invariant humanness ratings on one of the dimensions.

For the HN index, there was a significant main effect of objectification (lower in the appearance-focus condition), *F*(1, 165) = 4.39, *p* = .038, partial η^2^ = .026, but no main effect of provocation, *F*(1, 165) = 0.02, *p* = .894, partial η^2^ < .001, or interaction between provocation and objectification, *F*(1, 165) = 0.26, *p* = .612, partial η^2^ = .002. For the HU index, there were no significant main effects of objectification, *F*(1, 178) = 0.01, *p* = .930, partial η^2^ < .001, or provocation, *F*(1, 178) = 0.48, *p* = .491, partial η^2^ = .003, and no interaction, *F*(1, 178) = 2.52, *p* = .114, partial η^2^ = .014.

For mental agency, there were no significant main effects of objectification, *F*(1, 185) < 0.01, *p* = .998, partial η^2^ < .001, or provocation, *F*(1, 185) = 0.31, *p* = .579, partial η^2^ = .002, but there was a significant interaction, *F*(1, 185) = 4.24, *p* = .041, partial η^2^ = .022. For mental experience, there were no significant main effects of objectification, *F*(1, 185) = 0.30, *p* = .582, partial, η^2^ < .001, or provocation, *F*(1, 185) = 0.01, *p* = .913, partial η^2^ < .001, but as with agency there was a significant two-way interaction between provocation and objectification, *F*(1, 185) = 5.19, *p* = .024, partial η^2^ = .027. Simple effects analysis revealed that experience was attributed less to the partner in the objectification condition, but only in the absence of provocation.

For moral agency, there were no significant main effects of objectification, *F*(1, 185) = 0.89, *p* = .348, partial η^2^ = .005, provocation, *F*(1, 185) = 0.15, *p* = .903, partial η^2^ < .001, or their interaction, *F*(1, 185) = 0.40, *p* = .531, partial η^2^ = .002. There were also no significant effects for moral patiency: objectification main effect, *F*(1, 185) = 0.61, *p* = .436, partial η^2^ = .003; provocation main effect; *F*(1, 185) = 0.91, *p* = .342; and interaction effect, *F*(1, 185) = 1.12, *p* = .291, partial η^2^ = .006.

For the VDT, there was a significant main effect of objectification, *F*(1, 185) = 3.91, *p* = .049, partial η^2^ < .021. Men who focused on their romantic partner’s physical appearance stabbed the voodoo doll with more pins than those men who focused on her personality. There was also a significant main effect of provocation, *F*(1, 185) = 4.17, *p* = .043, partial η^2^ = .022, whereby provoked men acted more aggressively towards their romantic partner than those who were not provoked. However, these main effects were not qualified by the expected objectification x provocation interaction, *F*(1, 185) = 0.34, *p* = .561, partial η^2^ = .002.

### Discussion

In Study 3 we examined experimentally whether men induced to objectify their current female romantic partner would judge her to lack aspects of personhood and be more likely to behave aggressively towards her, especially when provoked. The findings offer partial support for these predictions. Objectification and provocation both produced heightened aggression on a proxy behavioral task, although not in interaction, and objectification reduced the level of HN men ascribed to their partners, implying a tendency to see them as subtly inert and thing-like. However, the objectification manipulation did not reduce other indices of personhood or consistently have differential effects on them as a function of provocation. The findings therefore offer stronger support for the view that objectification via appearance focus influences aggressive behavior than that it attenuates the perceived mind, humanness, or moral status of women in intimate heterosexual relationships.

These findings only partially align with previous research showing that appearance focus manipulations induce objectification by diminishing perceived humanness [14, 15, 20], mind [16, 19], or moral status [18, 19, 116]. However, our research is novel in examining these processes in romantic relationships rather than in men’s perceptions of female strangers. It is plausible that the emotional closeness and interdependency of these relationships mitigate denials of personhood and animalistic perceptions of the sort obtained in earlier studies. It is also possible that our use of an objectification manipulation that primarily elicited beauty- rather than sexuality-related appearance focus may have made animalizing perceptions (e.g., denial of HU traits) less likely [20, 26].

Despite the general failure to replicate these effects in the present study, with the exception of effects on HN traits, our findings support the counterintuitive view that subtle forms of dehumanization can occur even in our most intimate relationships [41] and the feminist argument that objectification may be implicated in violence against women [9, 12]. Although most studies to date have addressed sexual aggression, recent evidence also demonstrates that objectification encourages non-sexual, physical aggression towards women [35, 117]. Our findings add further support to this emerging research, extending it into the domain of intimate relationships.

## General discussion

In three studies, we show that objectification of women is implicated in men’s violent behavior towards women in romantic relationships. Men with stronger tendencies to implicitly associate women with objects are more likely to report engaging in physical violence and sexual coercion towards their partners (Study 1), a finding that partially replicates for tendencies to associate their own partners with objects (Study 2). These links are not reducible to effects of hostile sexism, although that consistently predicts multiple forms of violent behavior (Studies 1 & 2). Although implicit objectification was not directly related to partner-directed violence perpetration in a proxy behavioral task, high objectifiers were especially likely to aggress when intensely provoked by a story of their partner acting in a way that triggered men’s jealousy and upset (Study 2). When men were induced to objectify their partners via an appearance focus manipulation, they became more likely to behave aggressively towards their partners on the proxy task, implying that objectification plays a causal role in IPV. The manipulation also led them to see their partner as lacking the fundamentally human traits that differentiate us from inanimate objects (Study 3).

The implications of this work are both theoretical and practical. Theoretically, our evidence for a role of implicit objectification in IPV supports the claims of feminist philosophers that seeing women as objects is one contributor to violence against them. From the standpoint of psychological theory in particular, the findings indicate that objectification–understood either as a form of subtle dehumanization or as akin to one–can occur not only in relation to broad social categories but also towards specific individuals, including those within intimate relationships. Dehumanization-like phenomena occur between lovers as well as sworn enemies [122].

In this interpersonal context, our findings further suggest that the human-object distinction is more pertinent than the human-animal distinction that has been the focus of most research on dehumanization in intergroup settings. Seeing romantic partners as cold and unfeeling (object-like) may be more connected to IPV than seeing them as lacking rationality or sophistication or seeing them as animal-like. Although the great majority of research on intergroup dehumanization has emphasized animalistic forms, such as infrahumanization, in the domain of close relationships mechanistic or objectifying forms of dehumanization may be more consequential. Finally, the stronger evidence for the importance of nonconscious than conscious forms of objectification also implies that the roots of this process may be relatively deep-seated in culture and society rather than easily overcome by campaigns and exhortations. However, the strong evidence from Study 3 that appearance focus can facilitate violent behavior suggests one general target for interventions to reduce IPV. Preventive interventions that aim to reduce the cultural preoccupation with women’s appearance and cultivate a focus on their capabilities should undermine the implicit objectification that appears to underpin proneness to IPV perpetration. In addition, our finding that seeing their partners as unfeeling objects is implicated in heterosexual men’s tendency to engage in IPV could serve as a basis for therapeutic interventions with past or potential perpetrators.

Our studies have several strengths. They employ diverse methods, including two different implicit association measures and correlational as well as experimental designs, and are adequately powered to detect relatively subtle effects. Unlike most objectification research they complement self-report measures of aggression with a behavioral task. That task was personalized with the name of the female partner to make it real to participants, and included a narrative that added situational realism and was very successful in eliciting strong emotions from participants. At the same time, the studies also have limitations. Validity and reliability of the implicit measures may be relatively weak, impairing our capacity to detect relationships between IAT-based indices and other measures that are likely to be modest in magnitude given the many factors that contribute to IPV. The self-report measures of violence are likely to be imperfect records of actual recent behavior, and although the VDT is a well-established proxy measure of violent behavior that goes beyond self-report, it inevitably falls short of assessing actual instances of violent behavior towards partners rather than their digital avatars.

Despite these limitations, the studies point to objectification–understood as implicitly associating people with objects, as explicitly denying them mental states or fundamental human traits, or as a focus on a person’s appearance over their personhood–as an important psychological dimension of IPV. Further research is required to clarify the mechanisms through which objectifying perceptions of romantic partners facilitate or disinhibit physical, sexual, and psychological violence, and factors that contribute to these perceptions. Understanding why men behave violently towards those they profess to love is a high research priority, and the concept of objectification has an important role to play.
